# Systematic sampling of adults as a sensitive means of detecting persistence of lymphatic filariasis following mass drug administration in Sri Lanka

**DOI:** 10.1371/journal.pntd.0007365

**Published:** 2019-04-22

**Authors:** Ramakrishna U. Rao, Sandhya D. Samarasekera, Kumara C. Nagodavithana, Manjula W. Punchihewa, Udaya S. B. Ranasinghe, Gary J. Weil

**Affiliations:** 1 Infectious Diseases Division, Department of Internal Medicine, Washington University School of Medicine, St. Louis, MO, United States of America; 2 Anti-Filariasis Campaign, Ministry of Health, Nutrition and Indigenous Medicine, Colombo, Sri Lanka; 3 Regional Anti-Filariasis Unit, Galle, Sri Lanka; NIH-TRC-ICER, INDIA

## Abstract

**Background:**

Sri Lanka’s Anti-Filariasis Campaign conducted 5 annual rounds of mass drug administration (MDA) with diethylcarbamazine (DEC) plus albendazole to eliminate lymphatic filariasis (LF) in all endemic districts between 2002 and 2006. Post-MDA surveillance has consistently documented *Wuchereria bancrofti* microfilaremia (Mf) rates below 1% in all sentinel and spot check sites since that time, and all implementation units easily satisfied WHO’s target for school-based transmission assessment surveys (school-TAS) in 2013. However, more detailed studies have identified foci of persistent infection in the large coastal evaluation unit (EU) (population about 0.6 million) in Galle district. Therefore, the purpose of this study was to assess the sensitivity and feasibility of community-based TAS in adults (adult-TAS) and to compare results obtained by adult-TAS with prior school-TAS and molecular xenomonitoring (MX, molecular detection of filarial DNA in systematically sampled mosquitoes) results in this known problem area.

**Methodology and principal findings:**

Two cluster surveys were performed in independent samples of 30 evaluation areas (EAs) in the coastal Galle EU in 2015. Each survey tested approximately 1,800 adults for circulating filarial antigenemia (CFA) with the Alere Filariasis Test Strip. The CFA prevalence for all persons tested (N = 3,612) was 1.8% (CI 1.4–2.2), and this was significantly higher than the CFA rate of 0.4% obtained by school-TAS in 2013. CFA prevalences in the two samples were similar [1.5% (CI 1.0–2.2), and 2.0% (CI 1.4–2.7)]. Antigenemia prevalence in sampled EUs was highly variable (range 0–11%), and it exceeded 5% in 6 EAs. The 30 EAs sampled in one of our adult-TAS surveys had recently been assessed for persistent filariasis by molecular xenomonitoring (MX). CFA prevalence in adults and filarial DNA prevalence in mosquitoes in these EAs were significantly correlated (r = 0.43; *P* = 0.02).

**Conclusions:**

Community based adult-TAS provided a reproducible measure of persistent *W*. *bancrofti* infection in a large evaluation unit in Sri Lanka that has low-level persistence of LF following multiple rounds of MDA. In addition, adult-TAS and MX results illustrate the focality of persistent LF in this setting. Adult-TAS may be more sensitive than school-TAS for this purpose. Adult-TAS and MX are potential options for post-MDA and post-validation surveillance programs to identify problem areas that require mop-up activities. Adult-TAS should also be useful for remapping areas with uncertain LF endemicity for possible inclusion in national LF elimination programs.

## Author summary

Lymphatic filariasis (LF, also commonly known as “elephantiasis) is a leading cause of disability in the developing world. It is caused by parasitic worms that are transmitted by mosquitoes. The Sri Lankan Anti-Filariasis Campaign provided five annual rounds of MDA with diethylcarbamazine and albendazole in all infected areas between 2002 and 2006, and this reduced infection prevalence to very low levels in all sentinel and spot check sites. Sri Lanka conducted transmission assessment surveys (TAS, surveys for filarial antigenemia in primary school children) based on WHO guidelines in 2012–2013, and all evaluation units (EUs) in formerly endemic areas easily passed this test. However, other types of post-MDA surveys (antigenemia and microfilaremia assessments in communities, anti-filarial antibody assessment in primary grade school children and mosquito monitoring for filarial DNA) have shown evidence of persistent LF in Sri Lanka, with the strongest signals in a coastal EU in Galle district. Results from this study show that adult-TAS efficiently detected residual filarial infections in this EU that had passed school-TAS. Adult-TAS results were highly correlated with results from prior surveys that used molecular xenomonitoring (MX) to detect filarial parasite DNA in pools of mosquitoes collected in the same study areas. Thus adult-TAS and MX should be considered as an alternative surveillance approaches for verifying that LF has been eliminated following MDA and for identifying areas that require additional intervention.

## Introduction

The Global Programme to Eliminate Lymphatic Filariasis (GPELF) was initiated in 2000 with the goal of eliminating LF as a public health problem by 2020 [1–3]. GPELF uses mass administration of antifilarial medications (mass drug administration, MDA) to reduce microfilaria (Mf) prevalence below levels required for sustainable transmission by mosquitoes. As of 2017, the program had provided almost 7 billion treatment doses to some 890 million people, and this reduced the burden of disease in many countries [4–8].

WHO guidelines call for continuing annual MDA in implementation units (IUs) until Mf prevalence is below 1% in convenience samples of residents of sentinel and spot-check sites [3, 5]. This is followed by transmission assessment surveys (TAS, usually school-based cluster surveys) that are designed to show that the prevalence of infection in children ages 6 to 7 years in an evaluation unit (EU) is below 2% with 95% certainty in areas where LF is transmitted by *Culex* or *Anopheles* mosquitoes. TAS survey results are used to support decisions to stop MDA and for post-MDA surveillance [9–11]. While TAS provides information on recent transmission events in children, it has not been rigorously validated as a tool for detecting persistent LF in post-MDA settings. Recent post-MDA surveillance studies performed in Sri Lanka, India, American Samoa, and in Madagascar have documented significant persistence of filarial infection in adults and evidence for ongoing transmission in areas with <2% antigen prevalence in children [12–18]. School-TAS based on WHO guidelines employs children as sentinels for detecting recent transmission of the infection. However, this approach may provide inaccurate guidance in areas where children have less exposure to infection than adults. Another problem with the TAS strategy is that results from cluster surveys are assumed to be applicable across EUs with populations of up to two million people. Since LF prevalence is often highly focal, excellent survey results may be observed at the EU level despite persistence of LF in smaller areas within the EU.

The present study was performed to assess alternate approaches for detecting persistent LF in post-MDA settings with a focus on adult-TAS. Although adult-TAS is not useful for documenting recent transmission events, it should be superior to school-TAS for providing a measure of the persistent reservoir of filarial infection in EUs following MDA. If that is true, adult-TAS results may provide very useful guidance for LF elimination programs, because there can be no ongoing transmission without a reservoir of parasites in humans that be taken up by mosquitoes.

The study was performed in Sri Lanka, where bancroftian filariasis is transmitted by *Culex quinquefasciatus*. Sri Lanka’s Anti-Filariasis Campaign (AFC) was established in 1947, and it used a variety of approaches over the years to reduce filarial prevalence to low levels by 2002 [19, 20]. AFC then implemented five annual rounds of MDA with DEC and albendazole according to WHO guidelines in the years 2002–2006. The program achieved high coverage rates, and post-MDA surveys conducted between 2008 and 2013 showed that all EUs in the eight LF-endemic districts satisfied WHO criteria for elimination of LF as a public health problem; Mf prevalence was well below 1% in all sentinel and spot-check sites, and results of school-TAS easily met the WHO target [12, 20]. WHO officially recognized this achievement in 2016 with validation that Sri Lanka had eliminated LF as a public health problem [21]. However, comprehensive surveillance conducted between 2011 and 2016 that included assessments that were beyond those recommended by WHO demonstrated persistent foci of filarial infection (with high prevalences for Mf and filarial antigenemia and or antibody in humans and filarial DNA in mosquitoes) in some areas in the Southern province of Sri Lanka [12–14, 22]. The strongest signals for persistent LF were in a large coastal evaluation area in Galle district.

Galle district was a single implementation unit for MDA during the AFC’s LF elimination program [4, 20]. Following this program, in Galle Mf prevalences fell below 1%, and MDA was stopped [20]. AFC has conducted post-MDA surveillance activities since 2006. These included Mf surveys in sentinel and spot-check sites and extensive antigenemia surveys of primary school children in this district in 2008 and 2013 according to WHO guidelines active at those times. The survey in 2013 followed current WHO guidelines for TAS. Children were tested in 31 randomly selected schools per EU in 2 EUs. TAS results easily met the WHO target in these EUs [12]. However, separate surveillance activities by the AFC (“comprehensive post-MDA surveys” in two sentinel sites) plus district-wide molecular xenomonitoring (MX) surveys, and routine night blood surveys for Mf detected persistent LF in many areas in the coastal Galle EU [12–14, 22]. AFC provided an additional round of MDA with DEC plus albendazole in 2014 in 14 Medical Officer of Health (MOH) areas within the EU that were suspected to have persistent LF. The government’s estimate of MDA coverage was 73% (range 60–94%), and an independent assessment of epidemiological drug coverage (surveyed drug consumption) was 69% (range 62–80%) [23].

The present study evaluated the sensitivity and feasibility of community-based adult-TAS for detecting persistent LF at the EU level and compared those results with previously published school-TAS and MX results from this known problem area [12, 22].

## Materials and methods

### Study sites

Galle district (population 1.06 million) in the Southern Province of Sri Lanka is comprised of two EUs in Sri Lanka’s national LF elimination program. These include a coastal EU that was previously highly endemic, and an inland EU that historically had little LF. The coastal EU, with an approximate population of 609,000 and 124,600 households [24], is comprised of 12 MOH divisions that are comprised of some 210 smaller health administrative units called Public Health Midwife (PHM) areas. PHM areas were used as evaluation areas (EAs) for the cluster based adult-TAS described in this paper. The mean population of these EAs is approximately 6,700 (range 1200–8100). Three of the 12 MOH divisions included in this study belong to the inland EU of the district; they were included in this study, because they are adjacent to areas with known persistent LF in the coastal EU and because historical data show that they were endemic for LF in the past.

### Transmission assessment surveys in adults (adult-TAS) in the coastal Galle EU

The study was designed to determine whether community-based adult-TAS was sensitive for detecting residual filarial infections in the coastal Galle EU. The EU is comprised of 210 EAs. We conducted cluster-based sampling of residents in randomly selected EAs. Two independent surveys were performed to assess the reproducibility of the sampling protocol between May and October, 2015. Sampling was modified from the TAS protocol in Survey Sample Builder 2.3 software (http://www.ntdsupport.org/resources/transmission-assessment-survey-sample-builder). The first adult-TAS was performed in 30 randomly selected EAs that were previously surveyed by MX in 2014 [22]. The second survey was performed with an independent random sample of 30 EAs that were not in the first survey. Approximately 1800 adults (aged 18–92) from 30 EAs were screened for CFA in each survey. This sample size was similar to the number of children tested by school-TAS in this EU [12]. As with the school-TAS, if the number of positive antigen tests was less than the critical number of 18, that provided 95% confidence that the CFA prevalence in adult residents of the EU was less than 2%. The total numbers of HH (households) in the selected EAs in each of the two EUs were obtained from the government’s 2013 census list and assumed an average of 3 adults per HH. We estimated the target number of households (i.e. 600 HH) to survey by dividing the target sample size (n = 1800/EU) by the average size of the HH for adults. The number of HH in each of the 30 randomly selected EAs was estimated by dividing 600 HH with total number of EAs within that EU as approximately 20 HH per EA or 60 adults per EA. Fifteen to twenty households (HH) were systematically sampled in each EA. Maps, census data, and voter registries were used to identify HH locations. The sampling interval for houses was calculated by dividing the total number of houses in the EA by the number of HH to be sampled. Households from all 4 quadrants in each EA were included in the survey. A HH in the center of the EA was selected as the starting point, and subsequent HH were chosen by moving from the starting point in each of the 4 cardinal directions. In order to maintain consistency in sampling and to obtain geographically dispersed samples, no more than 4 persons were enrolled per household with no gender bias. Generally 3 or 4 HH were selected from each EA quadrant, and approximately 60 adults were enrolled in the study from each EA.

### Blood tests for *W*. *bancrofti* circulating filarial antigen

Alere Filariasis Test Strips (FTS, Alere Scarborough, Inc., Scarborough, ME, USA) were used to test 75 microliters of finger prick blood according to the manufacturer’s instructions. Two to three survey teams were deployed for each EA; each team included a public health inspector, a blood collector, a general assistant, and a supervisor. For CFA testing, blood samples were collected during the day with sterile, single use, contact-activated lancets (Fisher Scientific, Pittsburgh, PA). Finger prick blood was collected into plastic micropipettes and 75 μl of blood was added directly to sample application pads according to the manufacturer’s instructions. Tests were performed immediately after blood collection and read at 10 min. Test results were scored according to the intensity of the test “T” line relative to that of control “C” line as previously described [25]. Briefly, FTS scores were recorded as follows: 0, no “T” line visible; 1, “T” line is weaker than the “C” line; 2, “T” line is equal to the “C” line; and 3, “T” line is stronger than the “C” line. Results were entered into electronic case report forms that were preloaded onto smart mobile telephones.

### Detection of *W*. *bancrofti* microfilaremia

All individuals with positive CFA results were asked to provide blood samples between 21:00 PM and 23:00 PM for Mf testing. Finger prick blood was collected with capillary tubes into EDTA coated blood collection vials (Fisher Scientific). The next day, 60 μl of this blood was used to prepare 3-line thick blood smears [9, 12]. Once completely air dried, the smears were dehemoglobinized, stained with Giemsa, and examined by trained microscopists in the AFC laboratory in Colombo or in the Regional Filariasis Unit in Galle. Persons with negative CFA results were considered to be Mf-negative.

### Data collection and data management

Demographic information (EA, participant name, head of the household, age, gender, documentation of informed consent, reported bed net use the previous night, and prior MDA consumption) was entered into data collection forms in BLU mobile phones (Motorola Solutions, Inc., Schaumburg, IL) with Open Data Kit (ODK) LINKS software https://www.linkssystem.org [26]. Specimen ID and CFA test results were linked to participant identification numbers (QR-Quick Response codes). Unique identifiers were removed from data files prior to analysis. Lymphedema was recorded based on participant’s report as confirmed by the survey team.

GPS coordinates were collected for each studied HH, and HH locations were plotted using ArcGIS 10.5.1 (ESRI, Redlands, CA).

### Statistical methods

Deidentified, cleaned data were transferred into Microsoft Excel (Microsoft Corp., Redmond, WA) for descriptive analysis. Chi-squared or Fisher’s exact tests were used to assess the significance of differences in filariasis antigenemia prevalence. Filarial DNA prevalences in mosquitoes (maximum likelihood and 95% CI) that were estimated using Poolscreen 2.02 software in a previous study were used for analysis [22]. Correlations between human and mosquito infection parameters were assessed with the Spearman rank test using GraphPad Prism 7 software (La Jolla, CA). Graphs were produced with GraphPad Prism 7.

### Ethical approval

The human study protocol was approved by an ethical review committee at the University of Kelaniya (Gampaha, Sri Lanka) and by institutional review boards at Washington University School of Medicine and the Sri Lanka Ministry of Health. All participants in the study provided written informed consent. The AFC provided anti-filarial medications as per WHO guidelines to persons who had positive CFA test results.

## Results and discussion

### Antigen prevalence results

Sixty EAs were surveyed for *W*. *bancrofti* CFA and Mf prevalence. Approximately 3600 adults (age range 18–92, median age 44 years) from 1053 households (HH) living in 60 EAs were consented and enrolled in adult-TAS. 42.6% of these participants were males. Reported prior drug consumption during MDA campaigns was high (77%), and 68% of study participants reported bed net use the night prior to enrollment. A number of filarial lymphedema cases (44 of 3612, 1.2% of the population surveyed) were identified during the survey.

**Fig 1:**
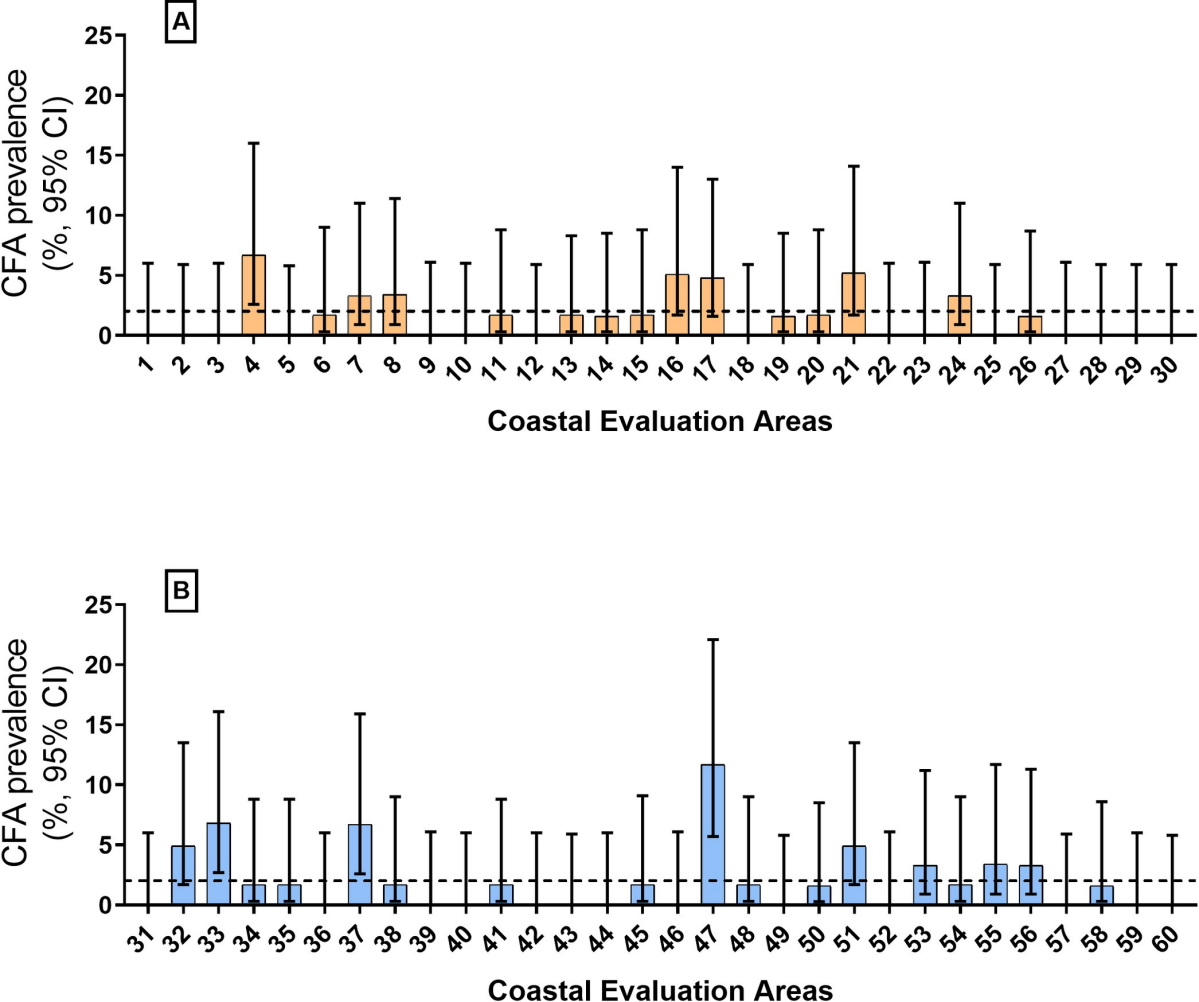
Summary of community circulating filarial antigen (CFA) prevalence in 60 evaluation areas (Public Health Midwife areas) assessed by adult-TAS in the coastal Galle evaluation unit. The antigenemia target of 2% (the upper CI for the prevalence estimate) for the EU is shown with a dotted line. The CFA prevalence was >2% in 7 of 30 evaluation areas in the first survey (panel A), and the prevalence > 2% in 8 of 30 EAs in the second survey (panel B).

Survey results are summarized in Table 1. Upper CIs for estimates of CFA prevalence exceeded the target value of 2% in seven of 30 EAs (23%) in the first survey (Fig 1A) and in 8 of 30 EAs (27%) in the second survey (Fig 1B). CFA prevalence estimates for the EU from these two surveys were not significantly different (Table 1). CFA prevalence estimates from 10 different randomly selected samples of 30 EAs selected from the pool of 60 EAs surveyed were quite similar (Table 1). The upper CI for the CFA prevalence estimates exceeded 2% for each of these EA samples. This suggests that the sampling protocol provided reproducible estimates of CFA prevalence in the EU. The estimated CFA prevalence exceeded 5% in 5 EAs (Patabendimulla in the Ambalangoda MOH area, Megalle in Galle MC, Palassa in Habaraduwa, Kalahe in Akmeemana, and in Piyagama in Balapitiya) (Fig 1A). The highest CFA prevalence observed was 11% in the Eththiligoda EA within the Galle Municipal Council (city limits) (Fig 1B).

**Table 1:** Community circulating filarial antigen (CFA) prevalence (%) results from adult-transmission assessment surveys (adult-TAS) in two different samples of 30 evaluation areas (EAs) in the coastal Galle evaluation unit.

Coastal Galle EU*	Survey Number	Population size	Number of EAs	Number of adults enrolled	CFA prevalence Number positive (%, 95% CI)
	I	111,809	30	1809	28 (1.5%, 1.0–2.2)
	II	106,295	30	1803	36 (2.0%, 1.4–2.7)
	Total	218,104	60	3612	64 (1.8%, 1.4–2.3)

*EU, coastal Galle Evaluation Unit (Population ~600,000). EA, Public Health Midwife area. Results from 10 different samples of 30 randomly selected EAs from the pool of 60 EAs provided similar results; the mean CFA prevalence (%) for 10 samples of 30 EAs was 1.85% ± SD 0.3%.

The CFA prevalence in EAs where at least one participant was CFA positive was not significantly lower in persons (n = 1418) who reported that they had ingested anti-filarial medications during the national MDA program in 2002–2006 [49 of 1418 (3.4%) vs. 14 of 494 (2.8%), *P* = 0.5]. These results are not surprising, because it is known that MDA with DEC plus albendazole does not always clear CFA. Also, some participants who reported that they had participated in MDA may have only swallowed one or two doses during the 5 rounds of MDA provided by the national program. It is also possible that study participants did not accurately recall their MDA participation history, because the program ended many years prior to our study.

As previously reported, school-TAS detected CFA in 7 of 1557 children (0.46%) in 31 randomly selected schools in 12 MOH areas in the coastal Galle EU in 2013 (S1 Table) [12]. This number was well below the threshold number of 18 required for the EU to fail TAS. Five of the seven positive children in that survey lived in the Balapitiya and Galle Municipal Council areas that were identified as problem areas in the current study (S1 Table).

The CFA prevalence in adults in this study was four times higher than the prevalence in children in the school-TAS (1.8% vs. 0.45%, P = 0.0003). There are several potential explanations for the increased sensitivity of adult-TAS for post-MDA surveillance in this setting. Prior studies have shown that the Alere FTS used for antigen detection in this study is more sensitive than the Binax NOW Filariasis card that was used in the school- TAS in 2013 [25, 27]. However, results from the test comparison study suggested that areas that pass school-TAS using the card test are also likely to pass if the FTS is used for antigen testing [27]. Secondly, young children (ages 6–7) may not be the right sentinel population for assessing residual LF and transmission in Sri Lanka and in other countries with similar characteristics. Children in Sri Lanka often receive albendazole (in school and/or at home) for deworming, and albendazole has significant antifilarial activity [28]. Young children also tend to be less exposed to mosquitoes than adults, because they spend less time outside in the evening. In addition, children may be more often protected by bed nets when they sleep.

**Fig 2:**
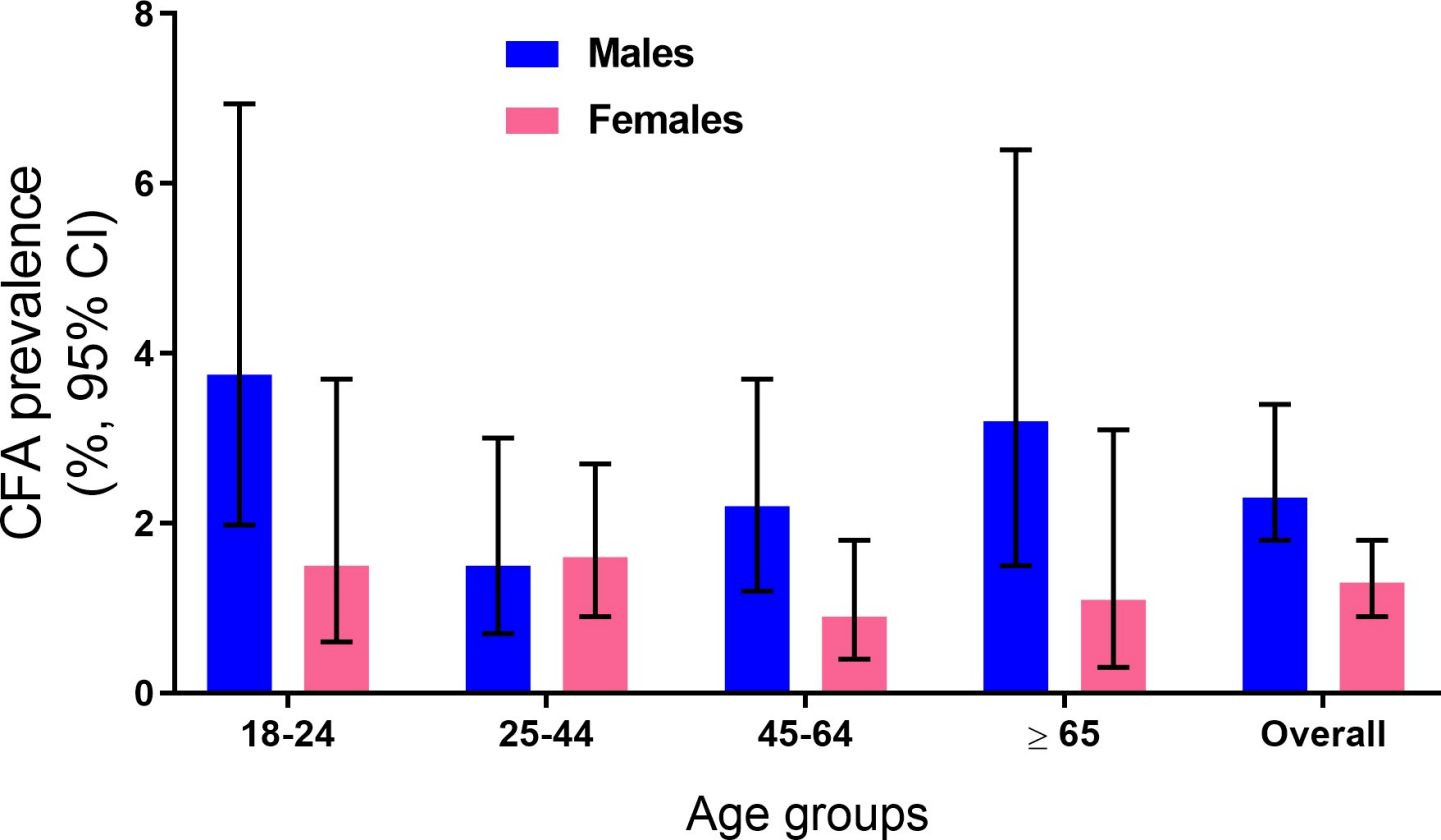
The figure shows circulating filarial antigen (CFA) prevalence (%) by age and gender for the population surveyed in 60 evaluation areas (EA) in the coastal Galle evaluation unit. Antigenemia was significantly more common in males for the total population (Males, 38 of 1537 or 2.5% vs. 26 of 2075 or 1.3% in Females, P = 0.007) and in most age groups.

CFA prevalence was much higher in males than in females (38 of 1537 or 2.5%, CI 1.8–3.4 *vs*. 26 of 2075 or 1.3%, CI 0.9–1.8, P = 0.007) (Fig 2). Thirty-three of 64 CFA positives (52%) had FTS scores of 1, and 31 (48%) had test scores ≥ 2. Relatively high rates of antigenemia in adults (especially in males) compared to prior TAS results in children may reflect their increased exposure to mosquitoes outside the home or to relatively low compliance in prior national MDA programs.

### Microfilaria test results

Night blood testing for Mf was performed for 62 of 64 (97%) study subjects who had positive CFA test results. Mf prevalence was very low with only two of 1809 (0.1%) in the first sample of 30 EAs and no Mf positives in the second EA sample. Only two of 62 (3.2%) participants with positive CFA results were Mf positive; both subjects were males (ages 43 and 68), both had FTS scores of 3, and their Mf counts were 39 and 8 by 60 μl thick blood smear, respectively. All 7 CFA positive children in the school-TAS performed in 2013 were Mf-negative [12]. Prior to the current adult-TAS, routine Mf testing conducted by the AFC found prevalences that ranged from 0% to 5% in coastal Galle EAs [14, 22]. The additional round of MDA that the AFC provided in these areas 6 months prior to the current study may partially explain the low Mf prevalence overall and the low Mf prevalence in persons with positive CFA results.

### Spatial distribution of CFA-positive households in adult-TAS and for schools included in the prior school-TAS

The map in S1 Fig shows locations for households surveyed for CFA in 60 EAs in the current adult-TAS study and for the 31 schools that were included in the school-TAS that was performed in 2013, two years prior to the this study. Fifty-nine of 1053 (5.4%) houses surveyed in the adult-TAS study had at least one CFA positive resident, and no more than 2 CFA positive residents were identified in any house. Some EAs included in the adult-TAS study had stronger CFA signals than others (S1 Table); percentages of HH with one or more CFA positive residents were 15% in the Galle Municipal Council area, 7% in Balapitiya, 5% in Ambalangoda, 9% in Habaraduwa, 7% Akmeemana and 5% in Hikkaduwa MOH areas. However, the map shows that CFA positivity was widely dispersed along the entire coastal area and not limited to a few foci.

### Comparison of CFA prevalence in humans and estimated prevalence of filarial DNA in *Cx*. *quinquefasciatus*

We previously reported results of MX surveys conducted in 30 EAs in the coastal Galle EU about 9 months prior to the adult-TAS study reported here [22]. The maximum likelihood of filarial DNA prevalence in mosquitoes exceeded the target (an upper confidence limit of 1% for filarial DNA in mosquitoes) in 12 of 30 EAs in the coastal EU at that time [22].

**Fig 3:**
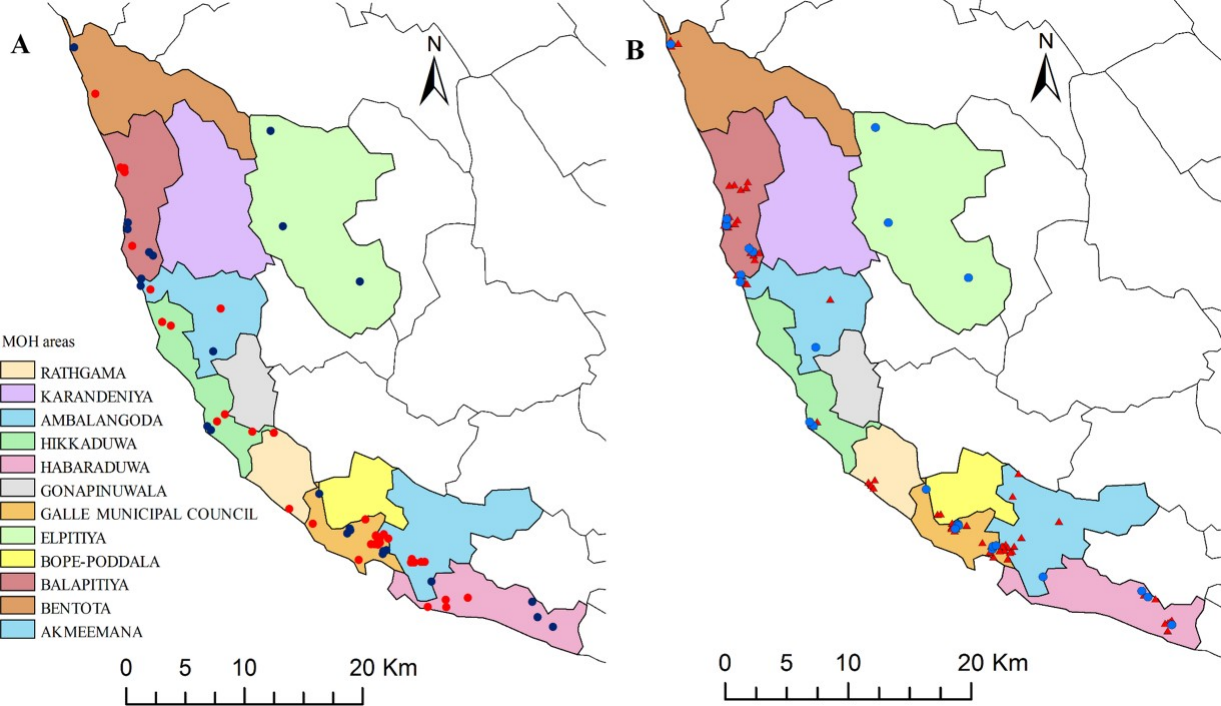
**The map shows locations with positive signals for persistent filariasis that were identified by adult-TAS antigenemia surveys (panel A) in 60 evaluation areas, and by adult-TAS and molecular xenomonitoring surveys (panel B) in 30 evaluation areas in the coastal Galle evaluation unit.** Panel A: Blue and red circles indicate households (HH) where at least one resident had a positive filarial antigen test in two surveys, respectively. Panel B: Blue circles indicate HH with positive signals for antigen in the first survey and red triangles indicate mosquito trap locations with one or more pools positive for *Wuchereria bancrofti* DNA. Infection signals in mosquitoes and in humans were widespread near the coast, but the strongest signals were detected in evaluation areas near Balapitiya and within the Galle Municipal Council (greater Galle city limits). Solid lines show the boundaries of Medical Officer of Health administrative areas within the EU in Galle district.

**Fig 4:**
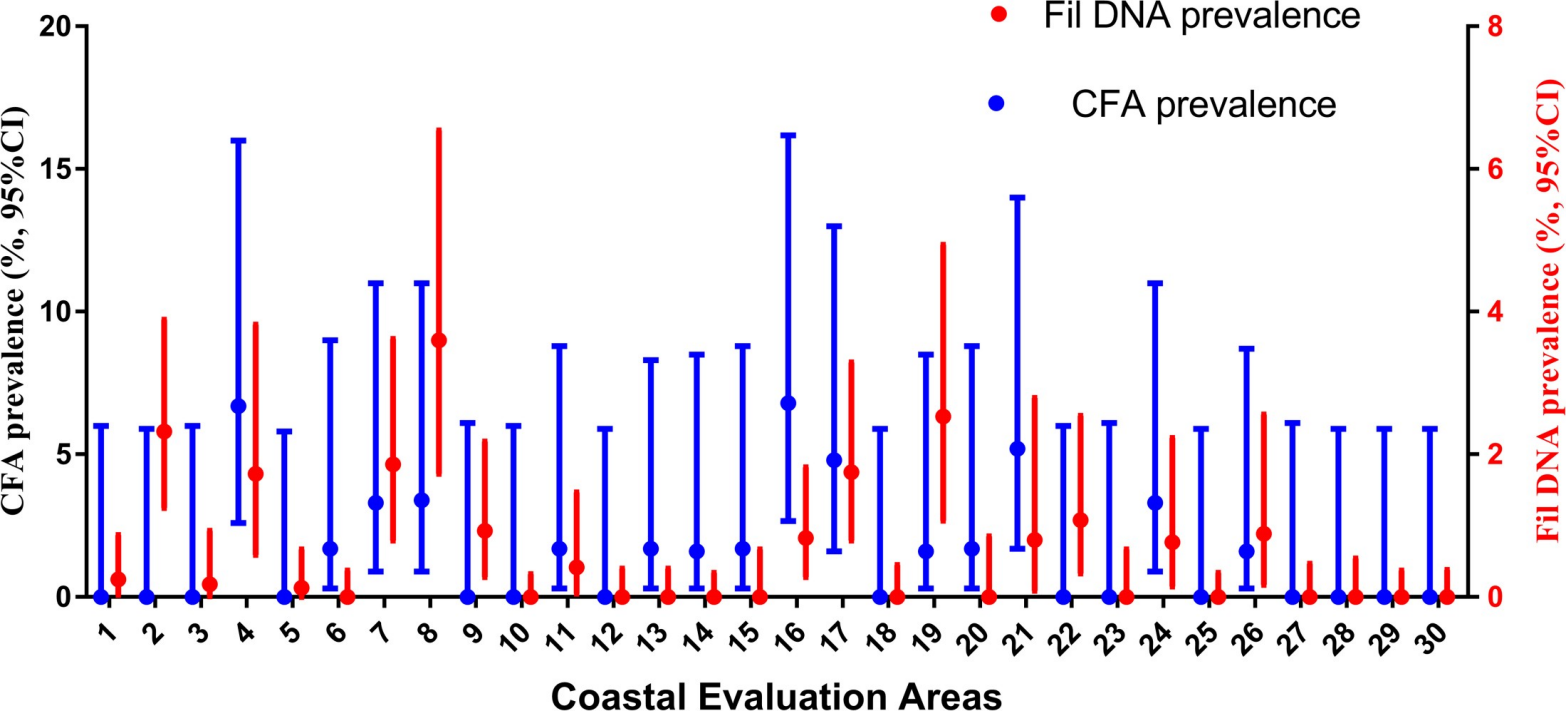
Comparison of circulating filarial antigenemia (CFA) prevalence obtained during adult transmission assessment surveys (adult-TAS, 2015) and filarial DNA prevalence in mosquito pools (molecular xenomonitoring survey-2014) in 30 evaluation areas in the coastal evaluation unit in Galle district. The association between human CFA prevalence and mosquito filarial DNA prevalence at the EA level was statistically significant (Spearman rank test, r = 0.43; *P* = 0.02).

Although sample sizes were small in individual EAs that were surveyed in the present study, CFA prevalence in the adult-TAS study exceeded the provisional target rate (an upper CI for prevalence of 2%) in 7 of 30 (23%) EAs in the first survey. Fig 3 shows locations of traps in 30 EAs where mosquitoes with filarial DNA were collected and the locations of HH with at least one CFA-positive resident in the coastal Galle EU. Results from these two types of surveys show persistent LF signals in many areas in the coastal Galle EU. CFA prevalence in adults and filarial DNA prevalence in mosquitoes in 30 EAs where both types of surveys were performed were significantly correlated (Spearman rank correlation, r = 0.4, P = 0.02, Fig 4).

The adult-TAS and MX results illustrate the focality of persistent LF in this post-MDA setting. The cluster sampling protocol for adult-TAS and school-TAS assumes that infection prevalence is either fairly uniform across EUs or that focality is not important. Adult-TAS appears to be more sensitive for detecting persistent LF than school-TAS in this post-MDA setting. Although the CFA prevalence in adults at the EU level was low (approximately 2%), many EAs in the EU had CFA prevalence >5%. This focality is a weakness of using TAS results in children or in adults as a firm endpoint for LF elimination programs.

## Additional discussion and conclusions

Adult-TAS results presented here and previously reported MX results from the coastal Galle EU show that both of these surveillance methods are more sensitive than school-TAS for detecting residual LF following MDA. MX is more sensitive than TAS but technically more difficult to perform. As noted in the Introduction, recent studies in India, American Samoa and in Madagascar where LF is transmitted by *Culex* have also documented significant persistence of LF in adults in areas that met school-TAS targets [15, 17, 18]. In addition to its value for post-MDA surveillance, adult-TAS may also be superior to the current practice of performing Mf surveys on convenience samples of adults in a small number of sentinel locations and for assessing the presence of LF in areas believed to be non-endemic that are adjacent to formerly endemic areas, because it samples more people in more locations.

This study in an EU with a population of ~600,000 in Sri Lanka and a recently published study with data from American Samoa (population: ~56,000) have shown that adult-TAS surveys are feasible for use by filariasis elimination programs [17]. However, community surveys are logistically more difficult than school surveys, because there are more places to visit and many adults are away from home during the day, and this can make it difficult to obtain representative samples of adult males and females. We recommend performing community surveys in the late afternoon or early evening and in the weekends or major holidays. The retrospectively collected cost data for conducting school-TAS in a variety of endemic settings is about $21,170 (median cost) including the cost of rapid diagnostic tests [29]. Additional studies are needed to assess the relative costs and benefits of different alternatives to school-TAS. These include adult-TAS, antibody-based school-TAS, and MX. Such studies should be performed in post-MDA settings with known low-level persistence of LF.

## Supporting information

S1 Checklist STROBE statement. Checklist of items. Rao et al., Systematic sampling of adults as a sensitive means of detecting persistence of lymphatic filariasis following mass drug administration in Sri Lanka.
(DOC)

S1 Table *Wuchereria bancrofti* infection prevalence in 12 Medical Officer of Health (MOH) areas in the coastal Galle EU by adult-TAS and school-TAS. (DOCX)

S1 Fig **The maps show distribution of all households in the coastal Galle evaluation unit that were tested for filarial infection by adult-TAS in 2015 (panel A) and schools during school-TAS in 2013 (panel B).** Red circles indicate households (HH) where at least one resident had a positive circulating filarial antigen (CFA) test result, and dark gray circles indicate locations of households with no CFA-positive residents (panel A). Schools with no CFA-positive children are shown with blue triangles, and schools with at least one CFA positive child are shown with pink triangles (panel B). An average of 32 adults were CFA positive in each sample of 30 EAs surveyed by adult-TAS. Only 7 of 1557 children were CFA positive in 31 schools surveyed by school-TAS. Five schools had at least one CFA positive child, and two of these schools had 2 positive children. Balapitiya, Galle Municipal Council, and Habaraduwa had many positive HH and at least one school with positive CFA test results. Boundary lines in the map show 12 MOH areas in the Galle coastal EU where 60 evaluation areas were surveyed for CFA in adults in the present study.
(TIF)
