# Supplementary material for: Systematic sampling of adults as a sensitive means of detecting persistence of lymphatic filariasis following mass drug administration in Sri Lanka
Source: PLoS Negl Trop Dis. 2019 Apr 22;13(4):e0007365. doi: 10.1371/journal.pntd.0007365 (PMC6497314; doi:10.1371/journal.pntd.0007365)
Supplement: S1 Table — (DOCX) [file pntd.0007365.s002.docx]

Table S1. *Wuchereria bancrofti* infection prevalence in 12 Medical Officer of Health (MOH) areas in the coastal Galle EU by adult-TAS and school-TAS

| MOH areas |  | Adult-TAS (2015 survey) | | | School-TAS (2013 survey) | |
| --- | --- | --- | --- | --- | --- | --- |
|  |  | Number of EAs surveyed | Average EA population  size (range) | CFA prevalence*  Positive/Total (%, 95% CI) | Number of Schools surveyed | CFA prevalence*  Positive/Total (%, 95% CI) |
| Akmeemana | | 6 | 3802 (2335-6084) | 7/362 (2, 0.9-4.0) | 2 | 0/62 (0, 0-0.5) |
| Ambalangoda | | 6 | 3337 (2545-4531) | 7/361 (1.9, 0.9-3.9) | 3 | 1/195 (0.5, 0-0.28) |
| Balapitiya | | 6 | 3413 (2260-4518) | 9/356 (2.5, 1.3-4.7) | 2 | 2/98 (2, 0.5-7) |
| Bope Poddala | | 4 | 3625 (3200-4270) | 2/240 (0.8, 0.2-2.9) | 2 | 0/135 (0, 0-2.7) |
| Elpitiya |  | 7 | 2835 (1848-3930) | 3/424 (0.7, 0.2-2.0) | 2 | 0/182 (0, 0-2.0) |
| Galle MC |  | 6 | 6804 (3470-8205) | 16/357 (4.5, 2.8-7.1) | 3 | 3/245 (1.2, 0.4-3.5) |
| Gonapinuwala | | 2 | 3109 (2960-3259) | 0/123, 0-3.0) | 1 | 0/10 (0, 0-27) |
| Habaraduwa | | 5 | 2946 (1993-3499) | 9/303 (3.0, 1.6-5.5) | 3 | 1/125 (0.8, 0.1-4.4) |
| Hikkaduwa | | 5 | 3491 (2818-4248) | 7/299 (2.3, 1.1-4.7) | 5 | 0/121 (0, 0-3) |
| Bentota/Induruwa |  | 5 | 3095 (2750-3382) | 2/304 (0.7, 0.2-2.4) | 3 | 0/141 (0, 0-2.6) |
| Karandeniya | | 4 | 3266 (1876-4380) | 0/244, 0-1.5) | 4 | 0/238 (0, 0-1.6) |
| Rathgama | | 4 | 3294 (1885-3803) | 2/239 (0.8, 0.2-3.0) | 1 | 0/5 (0, 0-43) |
|  |  | 60 |  | 64/3612 (1.8, 1.4-2.2) | 31 | 7/1557 (0.4, 0.2-0.9) |

TAS, Transmission Assessment Survey. EAs, evaluation areas. CFA, circulating filarial antigen. *, CFA prevalence (%) was determined by the Alere-FTS test for adult-TAS and by the Binax NOW Filariasis card test for school-TAS.
