# Supplementary figures and images for: Systematic sampling of adults as a sensitive means of detecting persistence of lymphatic filariasis following mass drug administration in Sri Lanka
Source: PLoS Negl Trop Dis. 2019 Apr 22;13(4):e0007365. doi: 10.1371/journal.pntd.0007365 (PMC6497314; doi:10.1371/journal.pntd.0007365)

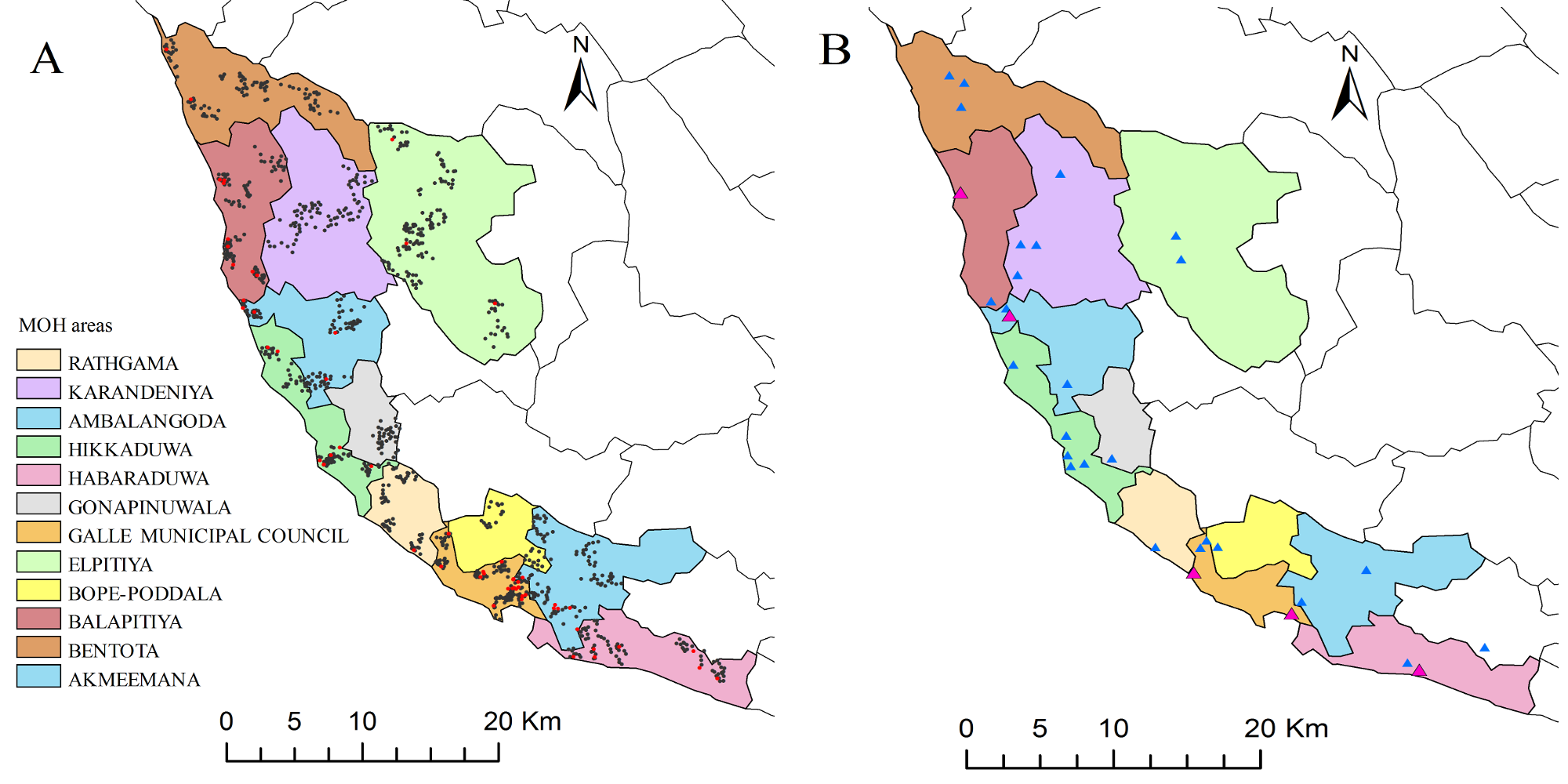

Supplement: S1 Fig — The maps show distribution of all households in the coastal Galle evaluation unit that were tested for filarial infection by adult-TAS in 2015 (panel A) and schools during school-TAS in 2013 (panel B). Red circles indicate households (HH) where at least one resident had a positive circulating filarial antigen (CFA) test result, and dark gray circles indicate locations of households with no CFA-positive residents (panel A). Schools with no CFA-positive children are shown with blue triangles, and schools with at least one CFA positive child are shown with pink triangles (panel B). An average of 32 adults were CFA positive in each sample of 30 EAs surveyed by adult-TAS. Only 7 of 1557 children were CFA positive in 31 schools surveyed by school-TAS. Five schools had at least one CFA positive child, and two of these schools had 2 positive children. Balapitiya, Galle Municipal Council, and Habaraduwa had many positive HH and at least one school with positive CFA test results. Boundary lines in the map show 12 MOH areas in the Galle coastal EU where 60 evaluation areas were surveyed for CFA in adults in the present study. (TIF) [file pntd.0007365.s003.tif]
